# An Overview on *Candida auris* in Healthcare Settings

**DOI:** 10.3390/jof9090913

**Published:** 2023-09-08

**Authors:** Maria Luisa Cristina, Anna Maria Spagnolo, Marina Sartini, Alessio Carbone, Martino Oliva, Elisa Schinca, Silvia Boni, Emanuele Pontali

**Affiliations:** 1 Department of Health Sciences, University of Genoa, Via Pastore 1, 16132 Genoa, Italy; cristinaml@unige.it (M.L.C.); am.spagnolo@unige.it (A.M.S.); elisa.schinca@unige.it (E.S.); 2 Hospital Hygiene Unit, E.O. Ospedali Galliera, 16128 Genova, Italy; 3 Infectious Disease Unit, Galliera Hospital, 16128 Genoa, Italy

**Keywords:** *Candida auris*, healthcare infections, antifungal resistance, epidemiology, prevention and control measures

## Abstract

*Candida auris* has become a major concern in critical care medicine due to the increasing number of immunocompromised patients and candidiasis is the most frequent cause of fungal infections. *C. auris* and other fungal pathogens are responsible for at least 13 million infections and 1.5 million deaths globally per year. In immunocompromised patients, infections can quickly become severe, causing wound infections, otitis and candidemia, resulting in high morbidity and mortality. The clinical presentation of *C. auris* is often non-specific and similar to other types of systemic infections; in addition, it is harder to identify from cultures than other, more common types of *Candida* spp. Some infections are particularly difficult to treat due to multi-resistance to several antifungal agents, including fluconazole (and other azoles), amphotericin B and echinocandins. This entails treatment with more drugs and at higher doses. Even after treatment for invasive infections, patients generally remain colonized for long periods, so all infection control measures must be followed during and after treatment of the *C. auris* infection. Screening patients for *C. auris* colonization enables facilities to identify individuals with *C. auris* colonization and to implement infection prevention and control measures. This pathogenic fungus shows an innate resilience, enabling survival and persistence in healthcare environment and the ability to rapidly colonize the patient’s skin and be easily transmitted within the healthcare setting, thus leading to a serious and prolonged outbreak.

## 1. Introduction

*Candida auris* has become a major concern in critical care medicine due to the increasing number of immunocompromised patients and candidiasis is the most frequent cause of fungal infections [1]. Patients hospitalized for COVID-19 are at risk for healthcare-associated infections (HAIs), including candidemia, or bloodstream infections caused by *Candida* [2].

Some isolates of *C. auris* can form aggregates both in vitro and in vivo that could allow it to evade the host’s immune system, escape attack by neutrophils, persist in host tissues and have a higher resistance to antifungals [3].

Various studies have described healthcare-associated infections caused by *C. auris* [4,5]. The aim of this narrative review is to describe *C. auris* contamination in healthcare settings, potential risks of infection, epidemiology, diagnostic and treatment, prevention and control measures.

To ensure a broad coverage of relevant literature, we undertook a MEDLINE search for English-language articles using the terms “*Candida auris*”, “healthcare facilities”, “antifungal resistance” and further relevant studies from reference lists of articles identified.

## 2. Characteristics of the Microorganism

*C. auris* is an opportunistic pathogen that can cause severe illness in hospitalized patients.

**Figure 1:**
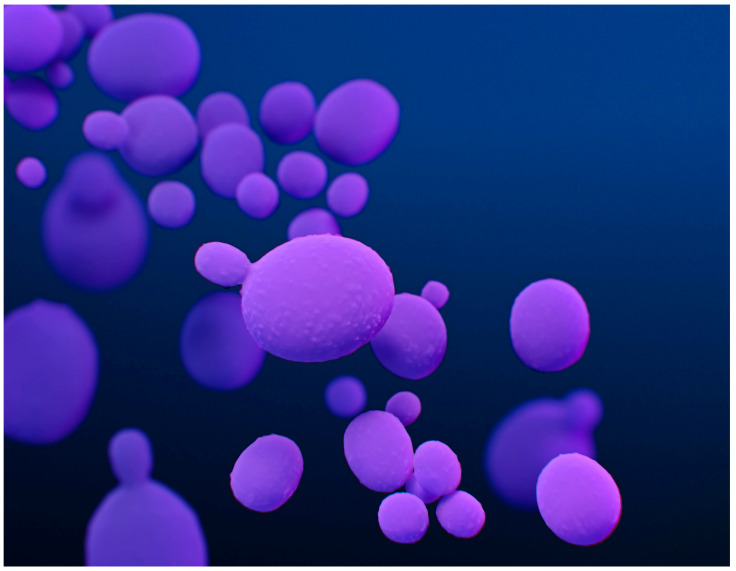
Morphology of cells of the microbial organism *C. auris* (Stephanie Rossow/Centers for Disease Control and Prevention—https://phil.cdc.gov/Details.aspx?pid=23239, accessed on 13 February 2023).

*C. auris* like other Fungi is a eukaryotic, heterotrophic, mainly aerobic organism, possessing chitin in its cell walls. It is a ubiquitous organism found in association with organic matter. *C. auris* is a haploid fungus with spherical or oval morphology with 2–3 × 2.5–5 µm cells, which normally grows as a yeast [6] unlike most other *Candida* species (Figure 1).

Recently, it was discovered that *C. auris* displays a phenotypic plasticity that allows this microorganism to hold a three-way phenotypic switching system. Strikingly, through this new system, *C. auris* can switch shapes among typical yeast, filamentation-competent yeast and filamentous form according to particular circumstances [6]. The latter morphology would be inhibited at the normal human body temperature of 37 °C and conversely favored at lower temperatures (26 °C). *C. auris* grows well at 40–42 °C and is able to survive in the environment and under alkaline conditions, although it cannot tolerate anaerobiosis. The transformation of *C. auris* from an environmental fungus to a human pathogen would be due to the overuse of antimycotics [7] or thermal adaptation due to climate change [8]. The hypothesis of an environmental origin would arise from the consideration that it is a fungus which is tolerant to high temperatures and hypersaline conditions before becoming pathogenic to humans [9]. The thermotolerance of *C. auris* has been proposed to be a characteristic that has allowed birds to transport the fungus across the globe to rural areas where humans and birds are in constant contact and from there it would quickly reach the urban environment up to healthcare facilities [10].

*C. auris* is phylogenetically related to *Candida haemulonii* and *Candida ruelliae* [11]. Four distinct clades (South American clade, African clade, South Asian clade and East Asian clade) have been identified from separate geographic origins, suggesting a recent and nearly simultaneous emergence of different clonal populations [12]. In 2018, an isolate representative of a potential fifth clade was identified in Iran, separated from the other clades by >200,000 single-nucleotide polymorphisms, susceptible to the three major classes of antifungal drugs. The isolate was cultured from ear swab specimens from a patient who had never travelled outside the country [13].

The virulence factors associated with *C. auris* infections are not completely understood. Probably the most important factor contributing to *Candida* virulence is the production of aspartyl proteinases (SAPS), enzymes involved in cell wall formation and adhesion, biofilm production, the ability to bypass the immune system and degradation and invasion of host tissues. The latter virulence factor would also be expressed by lipases. The ability of *C. auris* to express the various virulence factors is weaker than that of *C. albicans*, suggesting that *C. auris* is not as virulent as *C. albicans*. This decrease in virulence relative to *C. albicans* is likely due to the fact that *C. auris*, along with *C. glabrata* and *C. haemulonii*, is unable to develop hyphae or pseudohyphae in the mammalian host, which play critical roles in tissue invasion during infections [3].

## 3. Infections and Risk Factors

Patients may be colonized asymptomatically by *C. auris* on the skin, or in the nostrils, oropharynx, rectum and other body sites. However, unlike most other *Candida* species, which colonize the gastrointestinal tract, *C. auris* predominately colonizes the skin.

The interaction between skin commensal bacteria and fungi (microbiota) and *C. auris* can define its colonization level. Proctor et al. [14] investigated the associations between skin microbiota and *C. auris* colonization. The difference in both commensal bacteria and fungi communities present in *C. auris*-positive and *C. auris*-negative patients has provided evidence that the host microbiome may play an important role in the colonization of *C. auris* on the skin. Although microbiota dysbiosis was observed in the skin of *C. auris*-colonized individuals, it is still not known whether alterations in the microbial community are a consequence of *C. auris* colonization or whether microbiota dysbiosis contributes to *C. auris* colonization in the skin [15].

Patients with *C. auris* colonization can spread this yeast to other patients, and colonized patients can develop invasive as well as superficial infections. *C. auris* can cause severe illness and spreads easily among patients in healthcare facilities; it can cause candidemia, wound infections, otitis, and urogenital tract and respiratory tract infections. Antibiotic use disrupts the skin and gut microbiome, increasing *Candida* colonization and risk of invasive candidiasis [5].

Infections may be severe, and persistently positive blood cultures for >5 days or recurrent candidemia in those with *C. auris* candidemia have been reported [16].

Candidemias represent the most serious infections in patients with immunosuppression or major underlying diseases, such as diabetes mellitus, chronic kidney disease, HIV infection, solid tumors and hematological malignancies, and neutropenia; some cases have also been reported in newborns [17]. However, even in the absence of underlying disease, patients are at risk of invasive disease during intra-hospital outbreaks and depending on the ward concerned. Almost one third of patients with candidemia develop septic shock. In a study conducted by Bassetti et al. [18], age and abdominal source of the infection are the most important factors significantly associated with the development of septic shock in patients with candidemia.

Regarding extrinsic risk factors, a retrospective analysis of *C. auris* infections worldwide from 2009 to 2020 found that the five ones most often found in patients were a history of broad-spectrum antibiotic treatment (55.9%), central venous catheter (55.1%), intensive care unit (48.9%), urinary catheter (38.0%) and surgery (37.1%) [19]. Other risk factors include mechanical ventilation, parenteral nutrition, increased hospital length of stay, and residence in a high-acuity, skilled nursing facility. Infections have been observed several days to weeks after hospitalization in susceptible patients, suggesting exogenous sources of infection [20].

The patient and the environment can be reservoirs of fungal resistance in healthcare settings. *C. auris* studies have described widespread contamination of environmental surfaces and equipment persisting for months, with patient acquisition of *C. auris* occurring after as little as 4 h of contact. Ventilators in clinical care units were reported to spread *C. auris* infection [20].

This pathogenic fungus therefore shows an innate resilience, enabling survival and persistence in the clinical environment, and the ability to rapidly colonize the patient’s skin and be easily transmitted within the healthcare setting, thus leading to a serious and prolonged outbreak. In this context, the hands of healthcare personnel also play a crucial role with regard to the possible cross-transmission of *Candida* from colonized/infected patients and/or from contaminated surfaces to susceptible patients [21].

Several studies have shown the presence of various microorganisms including *C. auris* on different surfaces in healthcare environments [22,23], suggesting that these may be an important reservoir of contamination. *C. auris* was detected on the mattress, bedside table, bed rail, chair and windowsill in the room of a patient who had a *C. auris* bloodstream infection 3 months earlier and who remained persistently colonized in multiple body sites [24]. Contamination in the hospital environment has also been highlighted in other studies. During an outbreak in a London hospital, environmental sampling of the clinical area surrounding colonized patients demonstrated the contamination with *C. auris* of horizontal surfaces, such as the floor around bed sites, trollies, radiators, windowsills, equipment monitors and keypads, in addition to one air sample [25]. *C. auris* was also detected on reusable patient-monitoring equipment (axillary temperature probes and a pulse oximeter) and a patient hoist [26]. *C. auris* can also survive on non-porous plastic surfaces for at least two weeks, remaining metabolically active for at least 4 weeks [27]. Vallabhanei et al. [24] have highlighted how the persistence—weeks to months after initial infection—of colonization on skin and other body sites may represent a risk of contamination of the healthcare environment. In a surveillance study on *C. auris* colonization conducted by Ferrer Gomez et al. [28] in an ICU, it was found that of 124 colonized patients, 67% of pharyngeal carriers negativized at 1 month compared to 21% of cutaneous carriers, who negativized after 3–4 months. It has been shown experimentally that *C. auris* has a greater ability to survive on surfaces than *C. albicans*, but not compared with *C. parapsilosis* or *C. glabrata*. Compared with common bacterial pathogens, *C. auris* was detected with similar frequencies on dry surfaces and significantly more frequently on wet surfaces such as sinks. These results support the hypothesis that contaminated surfaces may be an important source of *C. auris* transmission [23].

*C. auris* is able to form biofilms on the surfaces it contaminates. Kean et al. [29] demonstrated that *C. auris* biofilms were more resistant to hospital disinfectants (chlorhexidine and hydrogen peroxide) than *C. albicans* and *C. glabrata* biofilms. Sherry et al. [30] demonstrated that *C. auris* can differentially adhere to polymeric surfaces, form biofilms and resist antifungal agents that are active against its planktonic counterparts. Of particular interest, caspofungin was predominately inactive against *C. auris* biofilms; this finding was unexpected because caspofungin is normally highly effective against *Candida* biofilms. These features contribute not only to *C. auris* virulence but also to its survival in hospital environments, increasing its ability to cause outbreaks.

## 4. Diagnosis

The clinical presentation of *C. auris* is often non-specific and similar to other types of systemic infections. Candidemia usually causes fever but no specific symptoms. Some patients develop a syndrome reminiscent of bacterial sepsis, with a fulminant course that may include shock, oliguria, renal failure and disseminated intravascular coagulation.

As *Candida* are commensals, their isolation from cultures of sputum, mouth, vagina, urine, faeces or skin does not necessarily indicate invasive and progressive infection. In addition, a characteristic clinical lesion must be present and histopathological tissue invasion documented, and other aetiologies must be excluded. Positive cultures of sample obtained from normally sterile sites, such as blood, cerebrospinal fluid, pericardial fluid or biopsy tissue provide definitive proof that systemic therapy is required [1].

Serum beta-glucan is often positive in patients with invasive candidiasis; in contrast, a negative result indicates a low probability of systemic infection.

Like other *Candida* infections, *C. auris* infections are diagnosed by culturing blood or other bodily fluids [1]. However, *C. auris* is harder to identify from cultures than other, more common types of *Candida*. For example, it can be confused with other types of yeasts, particularly *Candida haemulonii* [31].

Although new chromogenic media are available that further facilitate the identification of *C. auris*, the culture criterion cannot be the only method of identification [31]. From a laboratory point of view, the identification of *C. auris* is complex; in 2019, the Center for Disease Control and Prevention (CDC) developed an algorithm to identify *C. auris* based on phenotypic laboratory methods and initial species identification [32]. As a matter of fact, traditional phenotypic methods frequently misidentify *C. auris*. The proposed algorithm shows in detail the steps needed to determine the correct *Candida* spp. based on the tests and equipment available in laboratories (Table 1).

It is fundamental to apply phenotypic system and molecular techniques such as PCR and matrix-assisted laser desorption ionisation–time of flight (MALDI-TOF) platforms, VITEK2™ with the suitable updated databases and DNA sequencing [6]. Other identification methods include molecular tests such as DNA sequencing and WGS [9]. All *C. auris* strains from clinical isolation must be tested for susceptibility to antifungals to exclude therapy with drugs to which it is resistant.

## 5. Therapy and Antifungal Resistance

The Centers for Disease Control and Prevention do not recommend the treatment of *C. auris* isolated from non-invasive sites (e.g., respiratory tract, urine and skin) when there is no evidence of infection.

**Figure 2:**
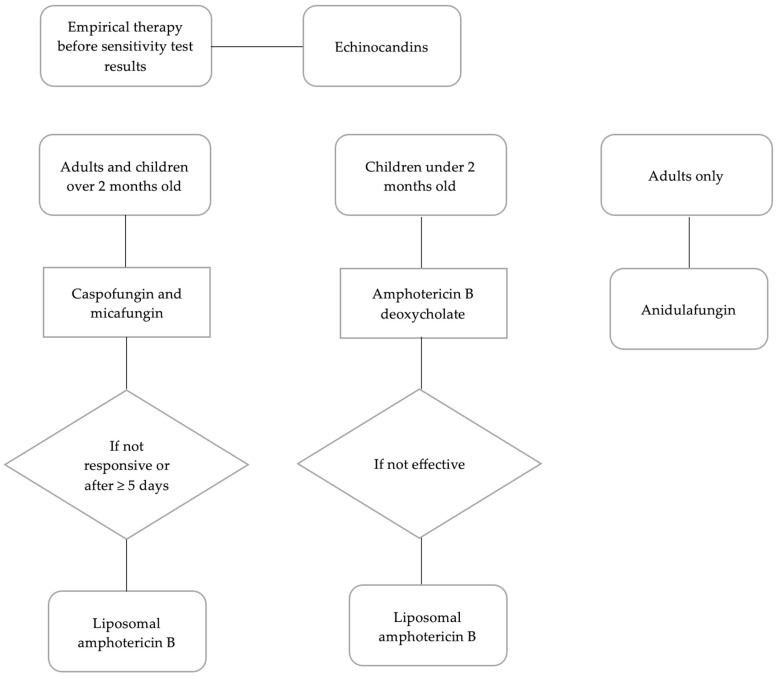
Flowchart of recommendations for treatment of *C. auris* infections ([33], modified).

Currently, azoles (e.g., fluconazole; itraconazole; isavuconazole; posaconazole; sertaconazole; voriconazole), polyenes (e.g., amphotericin B; nystatin) echinocandins (e.g., anidulafungin; caspofungin; micafungin) and nucleoside analogues (e.g., 5-flucytosine) are the main drugs used to treat fungal infections. The choice of antimycotic depends on the type and anatomical site of infection and fungal susceptibility. The CDC recommends the use of echinocandins as empirical therapy in the treatment of patients infected with *C. auris*, pending the availability of sensitivity test results [33]. Fluconazole and the echinocandins are the antifungal agents most used for the treatment of *Candida* bloodstream infection (candidaemia). Both are better tolerated than amphotericin B, which is less often prescribed due to the risk of toxicity [34]. Caspofungin and micafungin are recommended in the treatment of adults and children over 2 months of age, while anidulafungin is only recommended for adults. It is very important to follow the clinical course of these patients by repeating sensitivity tests to assess a possible acquisition of resistance. If the patient is not responsive to treatment with echinocandins or if the infection is persistent for more than five days, a switch to liposomal amphotericin B is recommended. In neonates and infants under two months of age, the initial choice of treatment is amphotericin B deoxycholate. If this is not effective, a switch to liposomal amphotericin B may be considered. Echinocandins may only be used in exceptional circumstances and only after ruling out central nervous system involvement (Figure 2) [33,35].

Some infections are particularly difficult to treat due to multi-resistance to several antifungal agents, including fluconazole (and other azoles), amphotericin B and echinocandins. This entails treatment with more drugs and at higher doses. In vitro, the interaction between voriconazole and micafungin showed an interesting synergy against multi-resistant *C. auris* strains. These promising results, however, were not found for the combination of other azoles with echinocandins [36].

There are currently no established *C. auris*-specific susceptibility breakpoint; therefore, breakpoints are defined based on those established for closely related *Candida* species and on expert opinion. The CDC attempted to establish breakpoints that could be considered as a general but not definitive guide to *C. auris* resistance patterns. Based on these MIC breakpoints, many isolates resulted as resistant to multiple classes of drugs. Some U.S. *C. auris* isolates have been found to be resistant to all three classes of antifungal drugs. The CDC received reports of pan-resistance found in other countries as well. According to the CDC’s latest upgrade, in the United States, about 90% of *C. auris* isolates have been resistant to fluconazole, about 30% have been resistant to amphotericin B and less than 5% have been resistant to echinocandins. These proportions may include multiple isolates from the same individuals and may change as more isolates are tested. The correlation between microbiologic breakpoints and clinical outcomes is not known at this time [37].

The mechanisms of molecular resistance involved in *C. auris* are many and differ depending on the class of antifungals being used. The mechanisms of resistance to azoles are mainly the overexpression of two efflux pumps: the ATP Binding Cassette (ABC) and the Major Facilitator Superfamily (MFS) transporters. One of the possible targets of azoles is the enzyme Lanosterol 14-alpha-demethylase (LD), encoded by the ERG11 gene, which converts lanosterol to ergosterol, a key component of the *C. auris* membrane. Point mutation or overexpression of the ERG11 gene has been shown to be responsible for reduced sensitivity to azoles. Echinocandins inhibit the enzyme Beta(1,3)D-glucan synthase which is encoded by the FKS1 and FKS2 genes, being composed of two subunits. Analyzing several isolates revealed numerous mutations in the FKS1 and FKS2 genes that confer resistance to this class of antifungals. Resistance to polyenes and in particular amphotericin B is mainly due to the modification of the sterol composition of the membrane. Finally, a molecular mechanism of resistance was also found among nucleoside analogues such as 5-Fluorocytosine. In a single Flucytosine-resistant isolate, an amino acid substitution (F2111I) was found in the FUR1 gene, which is involved in the metabolism of 5-Fluorocytosine. Especially for the latter classes of these drugs, given their reduced use compared to the others, little is still known about the molecular mechanisms that confer resistance to these fungi, so further research is needed [38].

In a recent 2023 study by Raschig et al., five new azobenzene derivatives were identified that were shown in vitro to inhibit growth in both fluconazole-sensitive and fluconazole-resistant *C. auris* isolates. These derivatives showed promising results both in terms of antifungal activity and cytotoxicity. Further in vivo studies are required to confirm this therapeutic potential [39].

In recent years, the increasing percentages of *C. auris* isolates resistant to the main classes of antifungals and the “high” mortality rate associated with it have posed a great challenge in the search for new therapeutic compounds and new approaches such as the use of nanoemulsions/nanoformulations that could complement or replace current antifungal therapies [40]. In a paper by Marena et al. [41] from 2023, nanoemulsions (NE) containing micafungin were developed to combat *C. auris* infections. Numerous in vitro evaluations were performed, including, for instance, the determination of the minimum inhibitory concentration (MIC). Nanoemulsions demonstrated efficacy both against biofilm and in an in vivo infection model, but not in planktonic cells. Here too, further studies are needed [41]. In another study from 2023 by Rosato et al., the in vitro efficacy of an essential oil of Cinnamomum cassia (CC-EO) both alone and formulated with polycaprolactone nanoparticles (nano CC-EO) was evaluated in 10 clinical strains of *C. auris*. Both compounds, both in free form and in nanoformulation form, showed interesting fungicidal activity and were therefore potential new resources in the fight against *C. auris* [42]. Recently, in the fight against disseminated candidiasis, the intravenous use of human immunoglobulins alone or in combination with amphotericin B have shown protective efficacy in prolonging the survival of mice with invasive candidiasis [43].

## 6. Epidemiology

*C. auris* and other fungal pathogens are responsible for at least 13 million infections and 1.5 million deaths globally per year, primarily in those with some compromised immune function [44]. *C. auris* has caused outbreaks on ICUs worldwide. It is the third most common cause of candidaemia in South Africa, with 88% of cases associated with ICU stays [5]. Due to lack of surveillance and limited capacity for laboratory detection, the worldwide prevalence of *C. auris* is likely to be underestimated [34].

Cases of *Candida* non-albicans spp. in the care setting have been found worldwide, presumably related to the prophylactic use of antifungal drugs in high-risk populations; however, *C. auris* appears to be unique in its ease of transmission between patients and in causing, therefore, outbreaks in the care setting and particularly in hospitals. In this respect, several molecular biology studies have confirmed intra- and inter-hospital transmission. It was first described in 2009 after being isolated from an external ear canal discharge of an inpatient in a Japanese hospital and since then has rapidly reported globally [11]. Cases of *C. auris* have been reported in more than 40 countries on six continents [34]. Because the identification of *C. auris* requires specialized laboratory methods, infections have likely occurred in other countries but have not been identified or reported [31].

*C. auris* has been recognized as majorly responsible for candidemia across the globe, surpassing the number of cases caused by *C. glabrata*, *C. tropicalis* and *Pichia kudriavzevii* in South Africa [2].

This organism poses a risk for patients in healthcare facilities due to its ability to cause infections in critically ill patients and its resistance to several antifungal agents, which makes infections difficult to treat. Patients hospitalized with severe COVID-19 are at risk of healthcare-associated infections, including candidaemia, and various outbreaks of *C. auris* among COVID-19 patients have been reported worldwide [34]. During 2021, the US reported over 3700 cases of *C. auris* colonization and over 1200 probable or confirmed cases.

**Figure 3:**
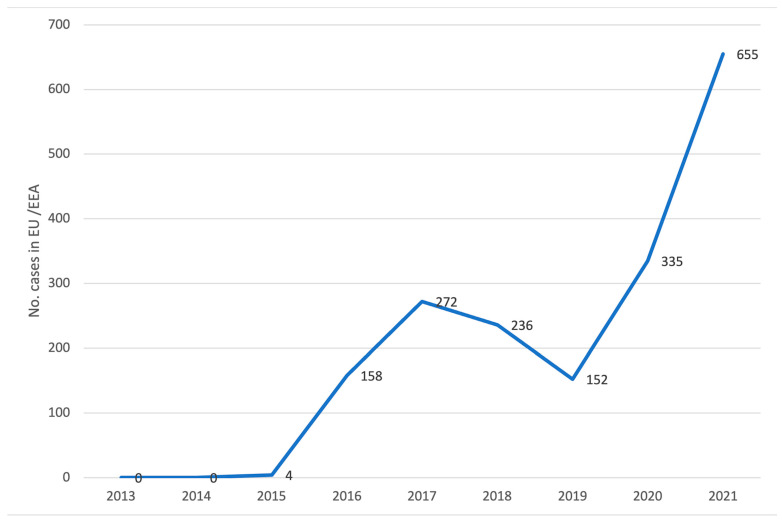
Reported cases of *C. auris* infection or carriage in thirty EU/EEA countries, 2013–2021 ([45], modified).

In Europe and the European Economic Area (EU/EEA), 670 cases were reported in the period January 2013—December 2018. In the period 2019 to 2021, another 1142 cases were reported (Figure 3) [45].

Moreover, between 2019 and 2021, five countries (Denmark, France, Germany, Greece and Italy) reported 14 *C. auris* outbreaks defined as two or more cases with an epidemiological link, with 327 affected patients in total. The number of patients affected per outbreak ranged from two to 214. Inter-facility transmission occurred in eight outbreaks, and three outbreaks were reported as ongoing at the time of the survey (Table 2) [45].

In Italy, the first case of invasive *C. auris* infection was identified in 2019, followed by an outbreak in northern regions in the pandemic period 2020–2021 [46]. In particular, in February 2020, *C. auris* was detected in an intensive care unit treating patients with severe COVID-19 in the same hospital, with a subsequent increase in case numbers throughout 2020 and 2021, and as of February 2022, a total number of 277 cases occurring in at least eight healthcare facilities in a region in northern Italy and a neighboring region [34].

The difficulty in its identification at laboratory level and the lack of knowledge of this species may delay early diagnosis, increasing the risk of horizontal transmission. According to data from the European Centre for Disease Prevention and Control, the number of *C. auris* cases reported in European countries has increased significantly in the last 2 years [34]. An increase in infections due to unidentified *Candida* species, including increases in isolation of *Candida* from urine specimens, should prompt suspicion of *C. auris* since *C. auris* can be transmitted in healthcare settings [31].

Since the start of the COVID-19 pandemic, outbreaks of *C. auris* have been reported in COVID-19 units of acute care hospitals. These outbreaks may be related to changes in routine infection control practices during the COVID-19 pandemic, including the limited availability of gloves and gowns, the reuse or extended use of these items and changes in cleaning and disinfection practices. Patients with COVID-19 who developed candidemia were less likely to have certain underlying conditions and procedures commonly associated with candidemia and more likely to have acute risk factors linked to COVID-19 care, including medicines that suppress the immune system [47].

The crude mortality rate in *C. auris* infections appears to be comparatively higher than for other species of *Candida*, ranging from 33% to 72% [48] and is associated with prolonged hospitalization (10–50 days), thus facilitating the spread of the pathogen in the hospital environment [28]. *C. auris* candidemia is associated with mortality rates of about 30–60%, depending on the setting [16]. However, as invasive *Candida* infections often occur in severely ill patients with multiple comorbidities, attributable mortality is difficult to determine [19,34].

## 7. Prevention and Control Measures

To decrease the risk of transmission in acute care settings, healthcare personnel should use standard and contact precautions as outlined by the CDC [49].

Even after treatment for invasive infections, patients generally remain colonized for long periods, so all infection control measures must be followed during and after the treatment of the *C. auris* infection [46].

If colonized or *Candida*-infected patients are to be transferred to other healthcare facilities, these must be informed of the presence of this multidrug-resistant microorganism to ensure that appropriate precautions are maintained.

Healthcare facilities must ensure thorough daily and terminal cleaning of rooms housing patients with *C. auris* infections, including the use of an EPA-registered disinfectant with an indication of efficacy against fungal microorganisms [24], such as chlorine-based disinfectants (at a concentration of 1000 ppm).

Cadnum et al. [50] demonstrated that hydrogen-peroxide-based disinfectants also have a high level of efficacy against different *Candida* species including *C. auris*, while quaternary ammonium compounds have relatively poor activity against all *Candida* species. UV-C devices could be useful as an adjunct to standard cleaning and disinfection to provide disinfection of any surfaces that are missed or inadequately covered by manual disinfection. In a study by Sherry et al. [30], chlorhexidine was found to be effective at a concentration of 0.02% in eradicating both planktonic and sessile cells of *C. auris*. The authors had underlined that the use of this disinfectant can be advocated for the topical control of *C. auris* at standard concentrations used for skin and wound cleansing and disinfection (0.05–4.0%) [30,51]. Biswal et al. pointed out that the adoption of multiple strategies was efficient against *C. auris*. In particular: elimination from colonized sites using chlorhexidine body and mouth washes; elimination from the environment using disinfectants; elimination from the hands of healthcare workers by training and improvements in hand hygiene compliance [51]. If possible, it is preferable to use disposable and dedicated equipment for each patient or cohort of patients with *C. auris* infection or colonization. Cleaning and disinfection of reusable equipment (e.g., monitoring devices, thermometers, pulse oximeters, blood pressure measuring instruments) according to manufacturer’s instructions should be ensured [34].

Although there is no evidence of a specific benefit of antimicrobial stewardship on the emergence and spread of *C. auris*, it is likely that an environment with a high use of broad-spectrum antibacterials and antimycotics favors the development of multi-resistant yeasts such as *C. auris*. Therefore, the implementation of antimicrobial stewardship, as well as it being an essential component of antimicrobial resistance reduction strategies, would mitigate the risk of *C. auris* spreading. The ECDC recalls the need to review prophylaxis in terms of risk–benefit analysis in settings with evidence of *C. auris* transmission [34].

## 8. *Candida auris* Surveillance Measures

Colonized patients may not only be able to transmit *C. auris* to other patients within healthcare facilities but may also be at risk of invasive infections. Screening patients for *C. auris* colonization enables facilities to identify individuals with *C. auris* colonization and to implement infection prevention and control measures [52].

According to the CDC, it is important to screen in those situations that place patients at high risk of contracting *C. auris*, such as close care contacts with patients with newly identified *C. auris* infection or colonization. The indication to carry out screening also applies to patients who have been admitted to a healthcare facility within the last year, especially if in a country with documented cases of *C. auris*, and to those who, admitted abroad, present infection or colonization with carbapenemase-producing Gram-negative bacteria as co-colonization of *C. auris* with these microorganisms has been observed regularly [53]. Screening is also recommended for patients who have had contact with a patient infected or colonized with *C. auris* (index patients) and for roommates in healthcare facilities, including nursing homes, where the index patient has resided in the previous month. Ideally, index patient roommates should be identified and screened even if they have been discharged from the facility. The CDC emphasizes that patients who require higher levels of care (e.g., mechanical ventilation) and who have stayed in the ward or unit with the index patient for 3 or more days should also be considered for screening as these patients are also at substantial risk of colonization. Patients with newly identified *C. auris* infection or colonization may have been colonized for months before the organism was detected. Therefore, when devising a screening strategy, it is also important to consider the patient’s previous health exposures and contacts [53]. Healthcare facilities should consider conducting more extensive screening, such as a point prevalence survey, if there is evidence or suspicion of ongoing transmission in a facility (e.g., *C. auris* detected in multiple patients through contact screening or clinical cultures, or increased infections with unidentified *Candida* species). Point prevalence surveys, especially when periodically repeated, yield a great deal of information on the size of the phenomenon and its trend over time, highlighting the potential effects of strategies adopted [54]. In a case-point prevalence survey, it is necessary to screen all patients in a given unit or floor where transmission is suspected and to consider performing a case-point prevalence survey even if all patients with known *C. auris* have been discharged. It is important to screen for *C. auris* colonization using a composite swab of the patient’s bilateral axilla and groin. Available data suggest that these represent the most common and recurrent sites of colonization. Although patients have been colonized by *C. auris* in the nose, mouth, external ear canals, urine, wounds and rectum, these sites are usually less significant for colonization screening [52].

Finally, surveillance systems for healthcare-associated infections should consider updating their definitions to include *C. auris* in the list of reportable pathogens linked with healthcare-associated infections [34].

## Figures and Tables

**Table 1:** Summary of CDC algorithm ([32], modified).

Identification Method	Database/Software, If Applicable	*C. auris* Is Confirmed If Initial Identification Is *C. auris*	*C. auris* Is Possible If the Following Initial Identifications Are Given. Further Work-Up Is Needed to Determine If the Isolate Is *C. auris*
Bruker Biotyper MALDI-TOF	RUO libraries (v2014 [5627] and more recent)	Yes	n/a
	CA System library (vClaim 4)	Yes	n/a
bioMérieux VITEK MS MALDI-TOF	RUO library (with Saramis v4.14 database and Saccharomycetaceae update)	Yes	n/a
	IVD library (v3.2)	Yes	n/a
	Earlier than IVD libraries (v3.2)	n/a	*C. haemulonii* *C. lusitaniae* No identification
VITEK 2 YST	Software version 8.01 *	Yes	*C. haemulonii* *C. duobushaemulonii* *Candida* spp. not identified
	Earlier than version 8.01	n/a	*C. haemulonii* *C. duobushaemulonii* *Candida* spp. not identified
API 20C		n/a	*Rhodotorula glutinis* (without characteristic red color) *C. sake* *Candida* spp. not identified
API ID 32C		n/a	*C. intermedia* *C. sake* *Saccharomyces kluyveri*
BD Phoenix		n/a	*C. catenulata* *C. haemulonii* *Candida* spp. not identified
MicroScan		n/a	*C. lusitaniae* ** C. guilliermondii ** C. parapsilosis ** C. famata *Candida* spp. not identified
RapID Yeast Plus		n/a	C. parapsilosis ** Candida spp. not identified
GenMark ePlex BCID-FP Panel		Yes	n/a

n/a: not applicable. * There have been reports of *C. auris* being misidentified as *C. lusitaniae* and *C. famata* on VITEK 2. A confirmatory test such as cornmeal agar may be warranted for these species. ** *C. guilliermondii*, *C. lusitaniae*, and *C. parapsilosis* generally make hyphae or pseudohyphae on cornmeal agar. If hyphae or pseudohyphae are not present on cornmeal agar, the isolate should raise suspicions of being *C. auris*, as *C. auris* typically does not make hyphae or pseudohyphae. However, some *C. auris* isolates have formed hyphae or pseudohyphae. Therefore, it would be prudent to consider any *C. guilliermondii*, *C. lusitaniae* and *C. parapsilosis* isolates identified on MicroScan and any *C. parapsilosis* isolates identified on RapID Yeast Plus as possible *C. auris* isolates, and further work-up should be considered.

**Table 2:** *C. auris* outbreaks in five EU/EEA countries detected in 2019–2021 (total of 14 outbreaks) ([45], modified).

Year *	No of Cases	Duration (Weeks)	Inter-Facility Transmission	Ongoing at Time of Survey
2019	214	149	Yes	Yes
2020	50	80	Yes	Yes
2020	15	44.5	Yes	No
2020	11	49	No	No
2021	10	5	Yes	No
2021	5	28	Yes	Yes
2021	4	3.5	Yes	No
2021	4	4	No	No
2021	3	4	Yes	No
2021	3	11	No	No
2021	2	8	No	No
2021	2	8	No	No
2021	2	39	Yes	No
2021	2	9	No	No
Total	327			

EEA: European Economic Area; EU: European Union. * year when the outbreak was detected, while the number of cases is reported for the whole duration of the outbreak.

## Data Availability

Data sharing not applicable.
